# Mesenchymal stem cells promote colorectal cancer progression through AMPK/mTOR-mediated NF-κB activation

**DOI:** 10.1038/srep21420

**Published:** 2016-02-19

**Authors:** Xiao-Bing Wu, Yang Liu, Gui-Hua Wang, Xiao Xu, Yang Cai, Hong-Yi Wang, Yan-Qi Li, Hong-Fang Meng, Fu Dai, Ji-De Jin

**Affiliations:** 1 Department of Gastroenterology, the Third Affiliated Hospital of Anhui Medical University, Hefei, Anhui 230061, P. R. China; 2 Institute of Radiation Medicine, Academy Military Medical Sciences, Beijing 100850, P. R. China; 3 The General Hospital of Chinese Armed Force Police, Beijing 100039, P. R. China; 4 The First Hospital Attached to Guiyang College of Traditional Chinese Medicine. Department of Clinical Laboratory, The First Hospital Attached to Guiyang College of Traditional Chinese Medicine, NO.71, Bao Shan North Road, Yunyan District, Guiyang City.

## Abstract

Mesenchymal stem cells (MSCs) exert a tumor-promoting effect in a variety of human cancers. This study was designed to identify the molecular mechanisms related to the tumor-promoting effect of MSCs in colorectal cancer. *In vitro* analysis of colorectal cancer cell lines cultured in MSC conditioned media (MSC-CM) showed that MSC-CM significantly promoted the progression of the cancer cells by enhancing cell proliferation, migration and colony formation. The tumorigenic effect of MSC-CM was attributed to altered expression of cell cycle regulatory proteins and inhibition of apoptosis. Furthermore, MSC-CM induced high level expression of a number of pluripotency factors in the cancer cells. ELISAs revealed MSC-CM contained higher levels of IL-6 and IL-8, which are associated with the progression of cancer. Moreover, MSC-CM downregulated AMPK mRNA and protein phosphorylation, but upregulated mTOR mRNA and protein phosphorylation. The NF-κB pathway was activated after addition of MSC-CM. An *in vivo* model in Balb/C mice confirmed the ability of MSC-CM to promote the invasion and proliferation of colorectal cancer cells. This study indicates that MSCs promote the progression of colorectal cancer via AMPK/mTOR-mediated NF-κB activation.

Mesenchymal stem cells (MSCs) reside in multiple organs and have been confirmed to contribute to tissue repair, and can be isolated and expanded for cell therapy^1^. However, therapy based on MSCs may be a double-edged sword, as MSCs have been demonstrated to play an important role in carcinogenesis by secreting high levels of cytokines that provide a supportive microenvironment for cancer cells^2^ and can even differentiate into cancer cells^3^. Preclinical data and animal models have demonstrated the involvement of MSCs as stromal cells that promote the initiation and development of colorectal cancer (CRC). Tsai *et al.* reported that MSCs can promote the formation of colorectal tumors in mice^4^. De Boeck *et al.* demonstrated that MSCs promote the invasion, survival and tumorigenicity of CRC cells *in vivo*^5^. A recent study indicated that MSCs promote CRC via very complex and multifaceted mechanisms^6^,^7^; therefore, the ability of MSCs to regulate the development of CRC requires further exploration.

Francipane *et al.* reported that excessive activation of the mTOR pathway leads to high level expression of downstream signal proteins that play important roles in the development of CRC^8^ and that targeting mTOR can induce apoptosis in CRC cells^9^. Gharibi *et al.* identified that the mTOR signaling pathway also promotes the growth of MSCs. Adenosine monophosphate-activated protein kinase (AMPK) acts upstream of mTOR to phosphorylate mTOR, which inhibits the activity of mTOR and promotes the growth of CRC cells in xenograft tumors^10^. Whether the AMPK/mTOR pathway plays a role in the ability of MSCs to promote CRC has not been reported.

The role of mTOR in the progression of cancer may also be related to define NF-κB^11^. NF-κB is an important nuclear transcription factor that is closely associated with the initiation and progression of CRC. NF-κB exists as dimer that most commonly contains the subunit P65 (RelA) and one of four other components^12^. Normally, dimerization of NF-κB is inhibited by IκB-α. Phosphorylation of IκB-α by the upstream kinases (I kappa B kinase [IKK]-alpha, IKK-beta, IKK-gamma and NF-kappa B-inducing kinase [NIK]), induces the subsequent ubiquitination of IκB-α, which leads to degradation of IκB-α and activation of the NF-κB pathway^13^.NF-κB can regulate the development of cancer as it transcriptionally activates a variety of apoptosis- and proliferation-related genes. It has been reported that multiple cytokines can excessively activate NF-κB and contribute to the genesis of cancer^14^,^15^. Thin *et al.* reported that MSCs secrete high levels of cytokines such as IL-6, which in turn downregulates the response of EC(endothelial cells) to inflammatory cytokines^16^. Whether MSCs promote CRC via activation of the AMPK/mTOR pathway remains to be studied, and it is unclear if NF-κB plays a role in the carcinogenic effect of MSCs via the AMPK/mTOR pathway.

This study aimed to identify the molecular mechanisms by which MSCs exert a tumor-promoting effect in CRC. We demonstrate that conditioned media from MSCs could promote proliferation, migration and colony formation and inhibit apoptosis in CRC cell lines. *In vivo* experiments confirmed that MSCs could promote invasion and metastasis in CRC. The effects of MSCs in CRC were mechanistically linked to activation of the AMPK/mTOR pathway and transcriptional activity of the NF-κB pathway. Collectively, these findings provide novel information on the mechanisms by which MSCs promote CRC.

## Methods

### Ethics and method statement

The present experiments including human and animal subjects were approved by the Ethics Committee of Academy Military Medical Sciences. All of the following protocols were approved in advance by the Academy of Military Medical Sciences, Beijing, China.

### Cell culture and preparation of conditioned medium

Studies involving human participants/subjects have been approved by ‘ review board of Ethics Committee of Academy of Military Medical Sciences, necessary consent from all the participants have been recorded. All investigations have been conducted according to the ethical principles suggested in the Declaration of Helsinki. Measures have been made to protect the privacy of research subjects and the confidentiality of their personal information. MSCs were isolated from bone marrow biopsies of patients without cancer, as previously described^17^. Briefly, bone marrow cells were flushed out of the bone cavities and then passed through a 70 μm cell strainer to obtain a single cell suspension. Mononuclear cells were prepared by Ficoll-Hypaque (Sigma-Aldrich, St Louis, MO, USA) gradient centrifugation. The single cells were seeded at 1 × 10^6 ^cells/dish in 100 mm culture dishes. At 1 day after seeding, the cells were cultured in α-MEM (Invitrogen, Carlsbad, CA, USA) supplemented with 10% FCS (HyClone Laboratories, Logan, UT, USA). After 1 week of culture, the cells had formed colonies and the adherent cells within the colonies were detached using trypsin, reseeded as new cultures, and expanded for further studies.

All cells were maintained in a humidified incubator at 37 °C and 5% CO_2_. MSC-conditioned medium (MSC-CM) was obtained from 1 × 10^6 ^MSCs. Cultures were washed three times with 10 ml PBS and incubated for 24 h at 37 °C in 10 ml α-MEM supplemented with 10% FCS. Control medium was collected in parallel from tissue culture flasks containing no cells. The medium was harvested and centrifuged at 1000 rpm for 5 min at 4 °C and the supernatant was stored at –20 °C. For the *in vivo* studies, the MSC-CM was concentrated 50-fold in ultrafiltration tubes with a 5 kDa Molecular Weight Cut Off (Millipore Billerica, MA, USA). HCT116 and LOVO CRC cell lines were routinely cultured in α-MEM supplemented with 10% FCS.

### Identification of MSC

The previously-described adherent cells were detached and labeled with CD19, CD34, CD73, CD90, CD45 and CD105 fluorescent conjugated antibodies (Becton, Dickinson and Company, NJ, USA). After evaluation by assessing the percentages of CD19(−), CD34(−), CD73(+), CD90(+), CD45(−) and CD105(+) cells, results of evaluation are displayed in Supplementary Figure S1A. The adipogenic, osteogenic and chrondrogenic differentiation abilities of the cells were also assessed in Figure S1C. For the adipogenic assay, 10000 MSCs were seeded into 24-well plates (per well) and cultured in DMEM (Sigma) containing 10% FBS and 100 mm/L indometacin, 10 μg/mL insulin, 0.5 mmol/L IBMX and 100 nmol/L dexamethasone (all Sigma) for every 3 day, then the cells were then stained with Oil Red O. For the osteogenic assay, 5000 MSCs (per well) were seeded into 24-well plates and cultured in DMEM (Sigma, City, CA, USA) containing 10% FBS and 0.1 μmol/L dexamethasone, 50 μmol/L ascorbic acid and 10 mmol/L β-glycerophosphate (all Sigma). After 4 weeks, the cells were stained with alizarin red (Sigma).For the chrondrogenic assay, 10000 MSCs were seeded into 24-well plates (per well) and cultured in DMEM (Sigma) containing 10% FBS and 10 ng/mL TGF-β, 10 μg/mL insulin and 100 nmol/L dexamethasone (all Sigma) for every 3 day, then the cells were then stained with toluidine blue.

### Proliferation assays

Equal numbers of HCT116 or LOVO cells were seeded into 96-well plates with MSC-CM or control medium and cell number was evaluated every 24 h for 6 days using the MTT assay, as previously described^17^. Absorbance was measured at 595 nm using a μQuant plate reader (Thermo Varioskan Flash, MA, USA).

For the BrdU assay, cells were seeded in 6-well plates and cultured in MSC-CM or control media for 24 h and then 10 μM BrdU (BrdU assay kit; Kai Ji, Nanjing, China) was added. After 7 to 8 h, cells were rinsed twice, incubated in working solution at 37 °C for 30 min, suspended in 200 mL staining buffer with 5 μl of PE-BrdU antibody for 30 min at 4 °C in the dark, and then imaged using an IX70 inverted microscope (Olympus, Hamburg, Germany). Experiments were performed in triplicate and tracks of at least 10 cells from three positions were analyzed in each well for each condition.

### Immunohistochemistry

HCT116 and LOVO cells were treated with MSC-CM or control medium for 24 h, and then fixed in 2% paraformaldehyde. The fixed cells or 5 μM-thick paraffin-embedded mouse colon sections were stained using antibodies against proliferation maker Ki67 (Bo Ao Seng, Beijing, China) or cell adhesion maker E-cadherin (Cell Signaling, Beverly, MA, USA). Briefly, the sections were dewaxed and rehydrated through a graded ethanol series, antigen retrieval was performed in 10 mM citrate buffer (pH 6.0) for 15 min in a microwave, then the slides were incubated with 3% hydrogen peroxide for 10 min (Solarbio, Beijing, China), blocked in 5% BSA for 1 h, incubated with the primary antibodies overnight at 4 °C, followed by incubation with biotinylated secondary antibodies conjugated to streptavidin-horseradish peroxidase (ZhongShan Golden Bridge Biotechnology, Beijing, China). Immunoreactivity was visualized using DAB solution. For the tissue sections, the average number of Ki67 and E-cadherin positive cells was determined from five consecutive sections from the tumor region.

### Transwell migration assay

The migratory behavior of HCT116 and LOVO cells that had been cultured with MSC-CM for 12 h was assessed using uncoated 8 μm pore-size Transwell inserts (Corning Incorporated, Corning, NY, USA). Briefly, 5 × 10^4^ viable cells were seeded per well, and MSC-CM or control medium were placed in the lower chamber. After incubation in 5% CO_2_ at 37 °C for 12 h, the cells were fixed in 2% paraformaldehyde in methanol and stained with crystal violet solution. The cells that had migrated to the bottom of the inserts were imaged under a light microscope and counted. All migration assays were performed in triplicate.

### Clonogenic assays

Briefly, 600 viable CRC cells were seeded into 12 well plates and treated with MSC-CM or control medium for 14 days to allow colony formation. Then, the colonies were fixed in 2% paraformaldehyde in methanol, stained with crystal violet solution and the numbers of colonies containing more than 50 cells were counted by light microscopy. Clonogenic assays were performed in triplicate.

### Soft-agar clonogenic assays

Soft-agar assays were performed to compare the clonogenic potential of CRC cells in semi-solid medium. Briefly, 6 × 10^2^ viable cells were suspended in 2 mL of MSC-CM or control medium containing 0.7% agar and plated on top of 2 mL of solidified 1.2% agar in 12-well plates. Plates were incubated at 37 °C for up to 14 days. The number of colonies was quantified under light microscopy using AnalySIS FIVE Image software. Each experiment was replicated three times.

### Cell cycle analyses

HCT116 cells were fixed in ice-cold 70% ethanol and incubated in 10 μg/mL propidium iodide solution containing 200 μg/mL RNase A. A BDAria II system (BD Biosciences, San Jose, CA, USA) was used for fluorescence-assisted cell sorting. For each experiment, 10000 events were counted, and cell cycle profiles were modeled using Modfit software (Verity Software House).

### Cell apoptosis assays

CRC cells were cultured in MSC-CM or control media at 37 °C for 24 h and apoptotic cells were detected using APC-conjugated JC-1 (BD Biosciences, San Jose, CA, USA). Briefly, after washing with cold PBS, 1 × 10^5 ^cells were resuspended in 100 μl JC-1 binding buffer with 5 μl of APC-conjugated JC-1 and then incubated for 15 min at room temperature. The numbers of apoptotic cells were determined by flow cytometry.

### ELISA assay

For the ELISA assay, 1 × 10^6 ^CRC cells were seeded into plates and cultured in 10 mL MSC-CM or control media for 24 h. The IL-6 and IL-8 concentrations of MSC-CM and the cell culture supernatants were determined using a commercially available ELISA kit (Xin Bo Sheng, Beijing, China) according to the manufacturer’s recommendations; IL-6 and IL-8 levels were calculated from the calibrator curves.

### Q-PCR

Total RNA was extracted from CRC cells using TRIzol Reagent (Invitrogen, Carlsbad, CA, USA). Real-time PCR was carried out on an ABI 7500 fast detection system using SYBR-Green I (Takara, Dalian, China). The relative expression of *P53, P16, P21, AMPK, mTOR, SOX-2, Oct4, C-myc* (compared to the house-keeping gene *GAPDH*) was quantified in separate tubes in triplicate using the following primers: *P53*: forward 5′-TGACTGTACCACCATCCACTA-3′ and reverse 5′-AAACACGCACCTCAAAGC-3′; *P16*: forward 5′-AACGCACCGAATAGTTACGG-3′and reverse 5′-CACCAGCGTGTCCCAGGAAG-3′; *P21*: forward 5′-TGTGATGCGCTAATGGCG-3′ and reverse 5′-AAGTCGAAGTTCCATCGCTCA-3′; *AMPK*: forward 5′-AACTGCAGAGAGCCATTCACTTT-3′ and reverse 5′-GGTGAAACTGAAGACAATGTGCTT-3′; *MTOR*: forward 5′-GTTCCGACGAATCTCAAAGC-3′ and reverse 5′-TCATATGTTCCTGGCACAGCC-3′; *SOX2*: forward 5′ -GCCGAGTGGAAACTTTTGTCG-3′ and reverse 5′-GGCAGCGTGTACTTATCCTTCT-3′; *Oct4*: forward 5′-CTGGGTTGATCCTCGGACCT-3′ and reverse 5′-CCATCGGAGTTGCTCTCCA-3′; *C-myc*: forward 5′-GGCTCCTGGCAAAAGGTCA-3′ and reverse 5′-CTGCGTAGTTGTGCTGATGT-3′ and *β-actin*: forward 5′-CCTGGCACCCAGCACAAT-3′ and reverse 5′-GGGCCGGACTCGTCATAC-3.

### Western blotting

Protein extracts were prepared and resolved on 12% SDS-PAGE gels. After transferring the proteins to nitrocellulose membranes, the membranes were blocked with 5% non-fat milk in PBS for 2 h and then incubated separately with primary antibodies against GAPDH, P53, AMPK, P-AMPK, mTOR, c-Myc, Sox-2, P-P65, P65, IKBα, P-IKBα (Cell Signaling, City, MA, USA), Bax or Bcl-2 (Boster Corporation, Wuhan, China) followed by incubation with horseradish peroxidase-conjugated secondary antibodies (ZhongShan Golden Bridge Biotechnology, Beijing, China). Antibodies were detected using enhanced chemiluminescence reagent (Pierce Biotechnology, City, IL, USA).

### NF-κB nuclear translocation and DNA binding activity assays

HCT116 cells were cultured in MSC-CM or control media for 24 h then nuclear proteins were extracted using the Nuclear Protein Extraction Kit (Sangon Biotech, Shanghai, China). Biotin-labeled probes for NF-κB (5′-AGTTGAGGGGACTTTCCCAGGC-3′ and 3′-TCAACTCCCCTGAAAGGGTCCG-5′) and the control OCT-1 binding site (5′-TGTCGAATGCAAATCACTAGAA-3′ and 3′-ACAGCTTACGTTTAGTGATCTT-5′) were generated using the EMSA probe biotin labeling kit (Beyotime, Nantong, China) and incubated with the nuclear extracts for 20 min in a total volume of 20 μl. The mixtures were separated by 4% non-denaturing polyacrylamide gel electrophoresis, transferred to nylon membranes and the probes were detected using a Chemiluminescent EMSA Kit (Beyotime, Nantong, China).

### Animal studies

Animal studies were carried out in accordance with the recommendations in the Guide for the Care and Use of Laboratory Animals of the National Institutes of Health (USA). All surgeries were performed under anesthesia with pentobarbital (40 mg/kg) to minimize suffering. Six-week-old Balb/c nude male mice (Wei Tong Li Hua, Beijing, China) were housed and maintained in a pathogen-free environment/barrier facility (Beijing Institute of Radiation Medicine, Beijing, China). Except for control group, mice were divided into 3 group with 6 mice in each group. The animals were intraperitoneally injected with 1 × 10^6^ HCT116 cells suspended in 200 μl of 50-fold concentrated MSC-CM or control medium. Mice were euthanized after five weeks, and then the colon tissues were excised for histological analysis. All animal procedures were approved by the Ethical Commission of the Beijing Institute of Radiation Science and the Ethical Commission Academy of Military Medicine Science, both of which approved the use of mice for this study (2014030)

### Histological examination

Colon tissues were fixed in 10% formalin, paraffin embedded and 5 μm-thick sections were prepared. Briefly, the sections were deparaffinized with xylene and redehydrated. Hematoxylin staining was performed for 5 min, followed by a wash in tap water and a 10 sec rinse in HCl solution (0.1%). After additional washing in tap water, the sections were stained in eosin solution for 2 min. Morphologic changes in the colon were examined and assessed using light microscopy. Each experiment was replicated by 6 times and analyzed by pathologist our lab.

### Statistical analysis

Data obtained from multiple experiments are reported as the mean ± SEM; significance was assessed using the Student’s *t*-test.

## Results

### Conditioned media from MSCs promotes the proliferation of CRC cell lines

**Figure 1:**
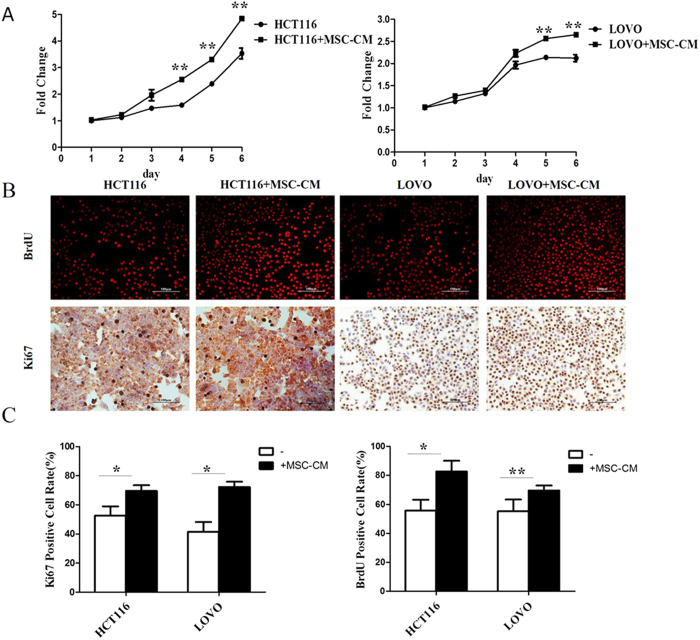
Conditioned media from MSCs promotes the proliferation of CRC cell lines. (**A**) HCT116 and LOVO CRC cells were cultured in MSC-CM or control media for 6 d, then cell proliferation was assessed using the MTT assay. (**B**) Representative images of HCT116 and LOVO CRC cells after culture in MSC-CM or control media for 24 h (100×). (**C**) The percentages of positive cells in the BrdU and Ki67 staining assays were determined by cell counting. *P < 0.05, **P < 0.01 vs. control group (n > 3). All photographs in this article were taken by Xiao-bing Wu.

To examine whether MSCs promote the proliferation of CRC cells, the MTT assay was performed on HCT116 and LOVO cells cultured in MSC-CM or control media. By day 5, culture in MSC-CM significantly increased the numbers of CRC cells compared to the cells cultured in control media (Fig. 1A). Due to the low sensitivity and small numbers of cells in the MTT assay, we used the BrdU assay to further confirm the effect of MSC-CM on CRC cell proliferation. The BrdU incorporation assay confirmed that culture in MSC-CM significantly increased the numbers of cells with active DNA synthesis compared to cells cultured in control media at 24 h (Fig. 1B,C). In further validation of these results, Ki67 staining (Fig. 1B,C) demonstrated that culture in MSC-CM significantly increased the proliferation of both CRC cell lines at 24 h.

### Conditioned media from MSCs increases the migratory and colony formation ability of CRC cell lines

**Figure 2:**
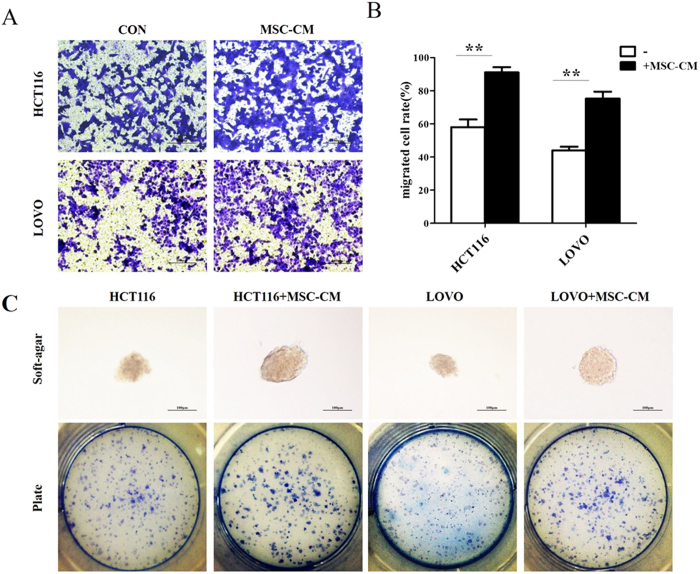
Conditioned media from MSCs increases the migratory ability and colony formation ability of CRC cell lines. (**A**) HCT116 and LOVO CRC cells were cultured in Transwell inserts in MSC-CM or control media for 12 h (100×). (**B**) The percentages of cells that migrated in the Transwell migration assay were determined by cell counting. (**C**) HCT116 and LOVO CRC cells were cultured in soft-agar or untreated plates with MSC-CM or control media for 14 d. **P < 0.01 vs. control group (n > 3).

The effects of MSC-CM on the migratory ability of HCT116 and LOVO cells were assessed using a modified Boyden chamber assay. Culture in MSC-CM significantly enhanced CRC cell migration compared to HCT116 and LOVO cells cultured in control media (Fig. 2A,B). The *in vitro* colony formation assay demonstrated that culture in MSC-CM significantly increased the colony formation ability of HCT116 and LOVO cells compared to cells treated with control media (Fig. 2C). These data indicate that MSCs can promote the migration and colony formation of CRC cells.

### Conditioned media from MSCs promotes the cell cycle and inhibits apoptosis in CRC cell lines

**Figure 3:**
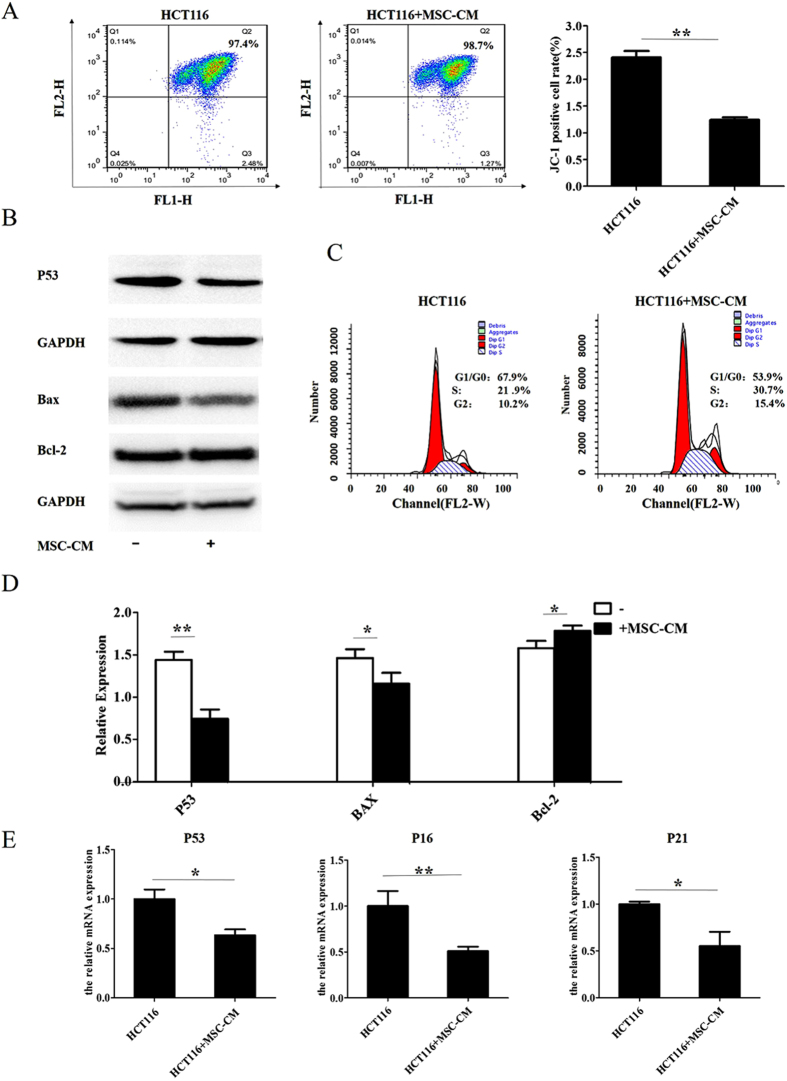
Conditioned media from MSCs regulates the cell cycle and inhibits apoptosis in CRC cell lines. HCT116 and LOVO cells were cultured in MSC-CM or control media for 24 h. (**A**) Flow cytometric analysis of JC-1 expression. (**B**) Western blot analysis of P53, Bax and Bcl-2 expression. (**C**) Flow cytometric analysis of cell cycle distribution in HCT116 cells. (**D**) Quantification of P53, Bax and Bcl-2 expression from Western blots. (**E**) Q-PCR analysis of P53, P21 and P16 mRNA expression. *P < 0.05 and **P < 0.01 vs. control group (n > 3).

The mitochondrial membrane potential assay was used to determine whether MSCs can promote CRC by inhibiting apoptosis. JC-1 staining was analyzed using flow cytometry and showed that culture in MSC-CM significantly decreased the rate of apoptosis in HCT116 cells compared with culture in control media (Fig. 3A). Western blotting demonstrated that the presence of MSC-CM downregulated the expression of the apoptosis-related proteins Bax and P53 and upregulated the anti-apoptotic protein Bcl-2 (Fig. 3B,D)compared to cells cultured in control media; the full-length blots are displayed in Supplementary Figure S2. To determine if the pro-proliferative effects of MSC-CM on HCT116 cells were associated with promotion of the cell cycle, we stained the cells with propidium iodine (PI) to assess the cell cycle using flow cytometry. MSC-CM significantly altered the cell cycle distribution of diploid CRC cells, with a tendency towards an increased percentage of cells in the S phase of the cell cycle (Fig. 3C). To further analyze the effect of MSC-CM on cell cycle progression, we also assessed the protein expression levels of two factors that negatively regulate the cell cycle. Western blotting demonstrated that HCT116 cells cultured in MSC-CM expressed significantly lower mRNA levels of both P53,P16 and P21 compared to cells cultured in control media (Fig. 3E). These results suggest that MSCs promote proliferation in CRC via a mechanism associated with altered cell cycle progression and inhibition of cell apoptosis.

### Conditioned media from MSCs contains cytokines and induces expression of pluripotency factors in CRC cell lines

**Figure 4:**
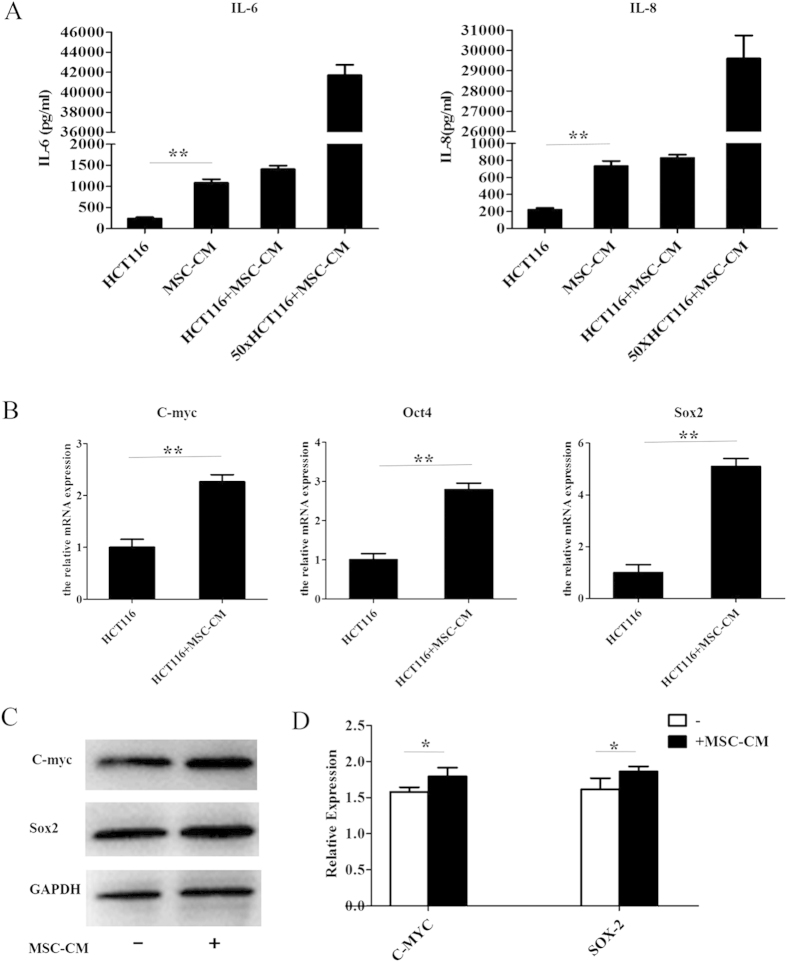
Conditioned media from MSCs contains cytokines and induces expression of pluripotency factors in CRC cell lines. (**A**) ELISA analysis of the levels of IL-6 and IL-8 in HCT-116 conditioned media, MSC-CM, concentrated MSC-CM (50×) and control media. (**B**) HCT116 cells were cultured in MSC-CM or control media for 24 h and the mRNA expression levels of C-myc, Sox2 and OCT4 were analyzed using Q-PCR. (**C**) Western blot analysis of c-Myc and Sox-2 protein expression. (**D**) Quantification of c-Myc and Sox-2 expression from Western blots. *P  < 0.05 and **P < 0.01 vs. control group (n > 3).

The results described above indicated that MSCs promote the proliferation and migration of cancer cells via a paracrine mechanism. To assess whether MSCs may promote CRC by secreting cytokines, we used ELISAs to analyze the levels of cytokines secreted by MSCs. MSC-CM contained significantly higher levels of IL-6 and IL-8 than control media (Fig. 4A). Additionally, HCT116 cells cultured in MSC-CM expressed significantly higher levels of *Sox2, Oct4* and *C-myc* compared to cells cultured in control media (Fig. 4B–D).

### Conditioned media from MSCs activates the AMPK/mTOR pathway in CRC cell lines

**Figure 5:**
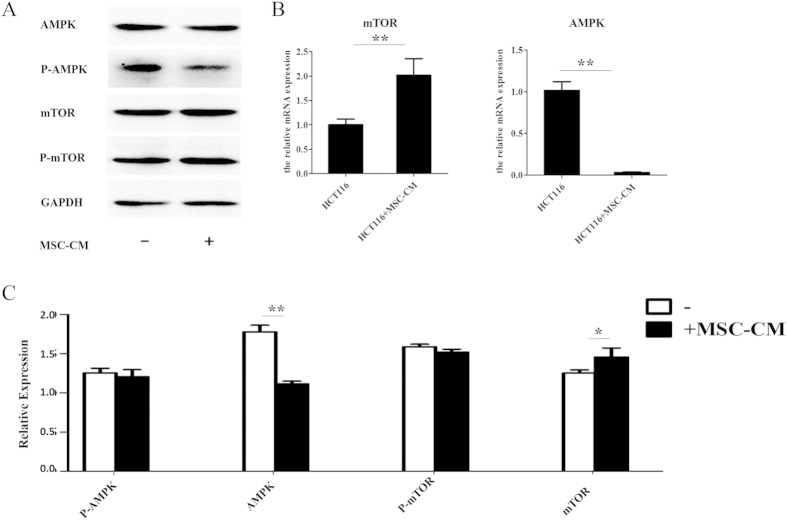
Conditioned media from MSCs activates the AMPK/mTOR pathway in CRC cell lines. HCT116 cells were cultured in MSC-CM or control media for 24 h. (**A**) The expression levels of AMPK, P-AMPK, mTOR and P-mTOR were evaluated by Western blot analysis. (**B**) The mRNA expression levels of AMPK and MTOR were determined by Q-PCR analysis. (**C**) Quantification of the protein expression of AMPK, P-AMPK, mTOR and P-mTOR. *P < 0.05 and **P < 0.01 vs. control group (n > 3).

To further investigate the mechanisms by which MSCs promote CRC, the phosphorylation of AMPK and mTOR were analyzed in protein lysates from HCT116 cells cultured in presence of control α-MEM or MSC-CM. Culture in MSC-CM did not significantly affect the overall expression of AMPK and mTOR (Fig. 5A–C), but significantly decreased the phosphorylation of AMPK and increased the phosphorylation of mTOR; full-length blots are shown in Supplementary Figure S2. Q-PCR confirmed that *AMPK* mRNA expression decreased and *mTOR* mRNA expression increased in cells cultured in MSC-CM (Fig. 5B). These results indicate that MSCs promote CRC via activating the AMPK/mTOR signaling pathway.

### Conditioned media from MSCs activates the NF-κB pathway in CRC cell lines

**Figure 6:**
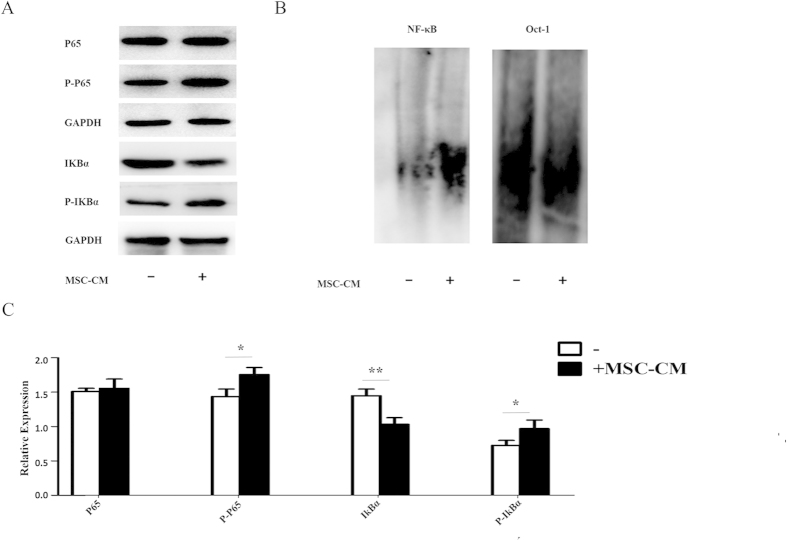
Conditioned media from MSCs activates the NF-κB pathway in CRC cell lines. HCT116 cells were cultured in MSC-CM or control media for 24 h (**A**) Western blot analysis of P65 expression, P65 phosphorylation, IKBα expression and IKBα phosphorylation. (**B**) NF-KB activity was determined using an EMSA; Oct-1 severed as a control. (**C**) Quantification of P65 expression, P65 phosphorylation, IKBα expression and IKBα phosphorylation. *P  < 0.05 and **P < 0.01 vs. control group (n > 3).

As described above, MSC-CM significantly increased the protein expression and phosphorylation of mTOR in HCT116 cells. The mTOR protein can affect the cell cycle, proliferation and differentiation by regulating a variety of cellular signals, including the NF-κB pathway. The NF-κB pathway contains several members, including the P65 subunit and its inhibitor IKBα. Western blotting demonstrated that culture in MSC-CM significantly increased the phosphorylation of P65 and IKBα in HCT116 cells (Fig. 6A,C) but did not alter the total expression of P65 (full-length blots are shown in Supplementary Figure S2.

An EMSA was used to further assess the effect of MSC-CM on NF-KB activity in CRC cells. MSC-CM significantly increased the transcriptional activity of NF-KB in HCT116 cells compared to cells cultured in control media; Oct-1 was used as a control (Fig. 6B). This data strongly suggests that MSCs promote CRC via activating the NF-KB pathway.

### MSCs promote tumorigenesis in a murine model of CRC

**Figure 7:**
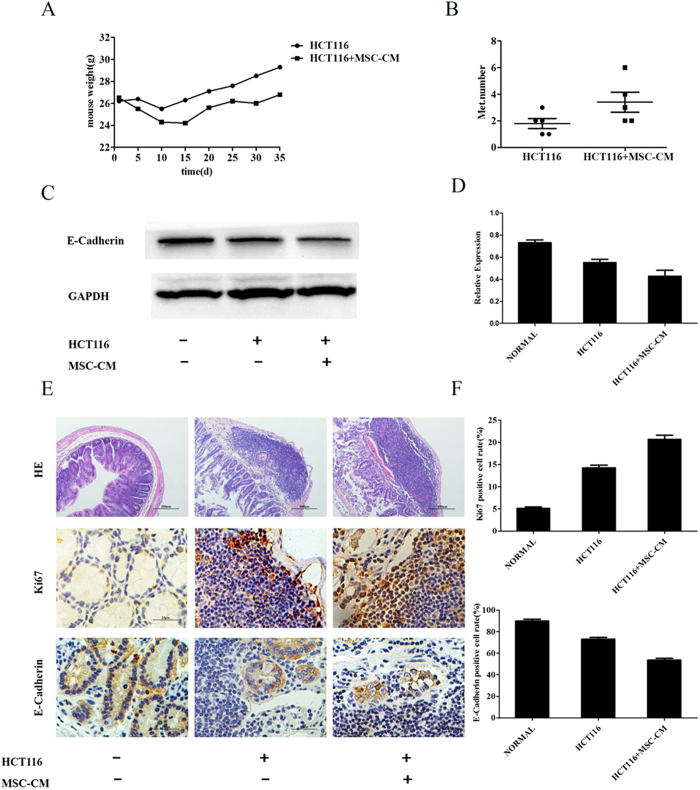
MSCs promote tumorigenesis in a murine model of CRC. BALB/C mice were intraperitoneally injected with HCT116 cells mixed with concentrated MSC-CM or control media. (**A**) The weight of the mice was measured every 5 days. (**B**) The mice were euthanized 5 weeks after injection of the cells, and their colons were excised and the numbers of peritoneal metastases were assessed. (**C**) Western blot analysis of E-Cadherin expression (100×). (**D**) Quantification of E-Cadherin expression. (**E**) Tumor invasion was assessed using H&E (40×) and immunohistochemistry for Ki67 or E-Cadherin (400×). (**F**) Quantification of the numbers of Ki67 or E-Cadherin positive cells in colon tissue sections.*P < 0.05 and **P < 0.01 vs. control group (n > 3).

For the *in vivo* assay, MSC-CM was concentrated 50-fold using ultrafiltration tubes. The concentrations of IL-6 and IL-8 in the concentrated MSC-CM are shown in Fig. 4A. Balb/C nude mice were intraperitoneally injected with HCT116 cells suspended in concentrated MSC-CM or control medium to establish a murine xenograft model of CRC. After 15 days, the weight of the mice injected with MSC-CM was significantly lower than the mice injected with control medium (Fig. 7A), and a higher number of mice in the MSC-CM group developed peritoneal metastases than the mice in the control group (Fig. 7B). Western blotting demonstrated that MSC-CM significantly decreased the E-Cadherin in a murine xenograft model of CRC. (Fig. 7C,D) Further E-cadherin, H&E and Ki67 staining (Fig. 7E,F) demonstrated that MSCs promoted the formation of CRC xenograft tumors, and also increased the extent of tumor invasion.

## Discussion

Bone marrow-derived stromal cells such as MSCs can migrate to primary tumors, indicating MSCs may have potential as candidate vehicles for the delivery of anti-cancer agents^18^. However, co-injection experiments have revealed that MSCs can also promote tumor growth and metastasis^4^, although the precise molecular mechanisms are not fully understood. To investigate whether MSCs intrinsically possess or acquire tumorigenic properties, we employed naive, multipotent MSCs derived from patients without cancer in functional *in vitro* and *in vivo* experiments with CRC cell lines. Our experiments demonstrated that bone marrow derived MSCs promoted the proliferation, invasion and tumorigenicity of CRC cells through activation of the AMPK/mTOR and NF-κB signaling pathways.

Although MSCs have been reported to development colorectal cancer^5^, the underlying mechanisms remain unexplored and the interactions between CRC and MSCs are complex. Therefore, we further investigated the influence of MSC-CM on CRC cell lines and the associated mechanisms in this study. The MTT and Ki67 and BrdU staining assays demonstrated that MSC-CM significantly enhanced the proliferation of CRC cell lines. Tumorigenicity and metastatic capacity, hallmarks of advanced tumors, can be induced by stromal cell contact or stimuli in many tumor cells including prostate cancer and head and neck cancer cell lines^19^,^20^. Likewise, our results revealed that MSC-CM significantly increased the tumorigenicity and metastatic capacity of CRC cells, as demonstrated by *in vitro* colony-formation and migration assays^21^. Moreover, the tumor-promoting effects of MSC-CM were demonstrated *in vivo,* as MSC-CM promoted tumor invasion and the formation of metastatic lesions in a mouse model of CRC. Taken together, these results strongly suggest that MSCs promote the progression of CRC.

MSCs are thought to promote cancer via a multifaceted mechanism. Rubio *et al.* reported that MSCs could regulate the cell cycle^22^ and play an important role in the granulocytic differentiation of acute promyelocytic leukemic cells^22^. In this study, cell cycle analysis demonstrated that culture with MSC-CM significantly increased the proportion of S phase cells, and decreased the mRNA and protein levels of P21 and P16, which are negative regulators of the cell cycle^23^, and downregulated the protein expression of the apoptosis-related protein P53. These results demonstrate that the ability of MSCs to promote the proliferation of CRC is linked to altered expression of cell cycle regulators.

This study indicates that the MSC-induced increases in the proliferation, survival, invasion and migration of CRC cells were due to cytokines secreted by the MSCs.

Numerous studies have suggested that MSCs promote angiogenesis, metastasis and other processes in cancer by secreting cytokines^24^,^25^,^26^,^27^. Additionally, it is well established that MSC secrete a variety of cytokines into the culture medium^28^. In this study, ELISAs demonstrated that MSC-CM contained high levels of IL-6 and IL-8, which can both activate NF-κB to promote cancer cell growth^29^,^30^. Additionally, Kansy BA demonstrated that Tumor-derived MSC constitutively produced high amounts of interleukin (IL)-6, IL-8 and stromal cell-derived factor (SDF)-1α, and can provided stromal support for human HNSCC cell lines *in vivo*^31^. Our results confirm MSC-CM induced high level expression of SOX-2, Oct4 and C-myc in CRC cell lines; these proteins are well-characterized tumor-associated pluripotency factors^32^. These results indicate that MSCs promote the progression of CRC by inducing a stemness phenotype^33^.

Although the ability of MSCs to increase the viability, migration and proliferation of CRC cells has previously been reported, the detailed molecular mechanisms remain unclear. Previous studies showed that AMPK and mTOR gene expression play a key role in inducing a metabolic shift and enhance the metastasis of CRC cells^34^,^35^,^36^. The current study also revealed that culture with MSC-CM downregulated the levels of phosphorylated AMPK in CRC cells. Both the mRNA and phosphorylation levels of mTOR, a molecule downstream of AMPK^37^, were upregulated in CRC cells cultured in MSC-CM. This indicates that activation of the AMPK/mTOR pathway plays an important role in the ability of MSCs to promote the progression of CRC. The role of the AMPK/mTOR pathway in the progression of cancer has also been related to NF-κB. Inhibition of mTOR can induce phosphorylation of the Ser32 and Ser36 residues of IKB-a, which in turns leads to activation of the NF-kB pathway^38^,^39^. Other studies have revealed that the nuclear transcription factor NF-κB exerts a tumorigenic effect in cancer^40^. This study showed that MSC-CM increased P65 and IKBα protein expression and upregulated NF-κB transcriptional activity, demonstrating that MSC-CM activates the NF-κB pathway in CRC cell lines. These results suggest that MSCs may promote the progression of CRC by activating the AMPK/mTOR and NF-κB signaling pathways in CRC cells.

In conclusion, this study demonstrates that MSCs stimulate the proliferation invasion, survival, tumorigenicity and migration of CRC cells in a paracrine manner. The tumorigenic effects of MSCs are mechanistically linked to activation of the AMPK/mTOR pathway and NF-κB pathway. Further exploration of these pathways will help to fully elucidate the tumorigenic effect of MSCs in CRC.

## Additional Information

**How to cite this article**: Wu, X.-B. *et al.* Mesenchymal stem cells promote colorectal cancer progression through AMPK/mTOR-mediated NF-κB activation. *Sci. Rep.* **6**, 21420; doi: 10.1038/srep21420 (2016).

## Supplementary Material

Supplementary Information
