# Supplementary material for: Mesenchymal stem cells promote colorectal cancer progression through AMPK/mTOR-mediated NF-κB activation
Source: Sci Rep. 2016 Feb 19;6:21420. doi: 10.1038/srep21420 (PMC4759824; doi:10.1038/srep21420)
Supplement: Supplementary Information [file srep21420-s1.pdf]

SUPPLEMENTARY INFORMATION TO

Mesenchymal stem cells promote colorectal cancer progression through AMPK/mTOR-mediated NF-κB activation

Xiao-bing Wu 1,2, Yang Liu2, Gui-hua Wang 2, Xiao Xu3,Yang Cai2,Hong-yi Wang2, Yan-qi Li2, Hong-fang Meng2, Fu Dai1*&Ji-de Jin2**

* Corresponding Authors

**Co-Corresponding author

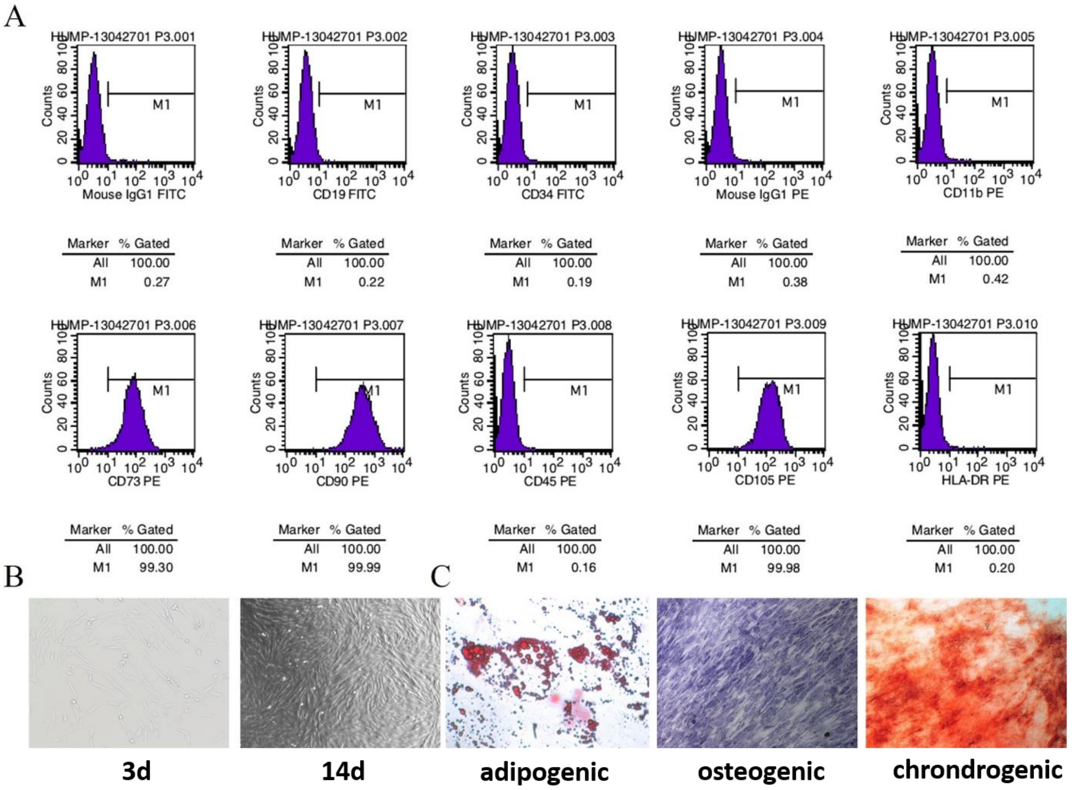

Supplementary Figure S1. Identification of Mesenchymal stem cells (MSCs). (A) cells were detached and labeled with CD19, CD34, CD73, CD90, CD45 and CD105 fluorescent conjugated antibodies and the percentages of CD19(-), CD34(-), CD73(+), CD90(+), CD45(-) and CD105(+) cells were detected by Flow cytometery. (B) Morphology of cultured MSC in day 3 and day 14. (C) MSCs were seeded into 24-well plates (per well) and cultured in adipogenic， osteogenic and chrondrogenic differentiation medium, after 4 weeks, cells were stained with Oil Red O, β- alizarin red and toluidine blue.

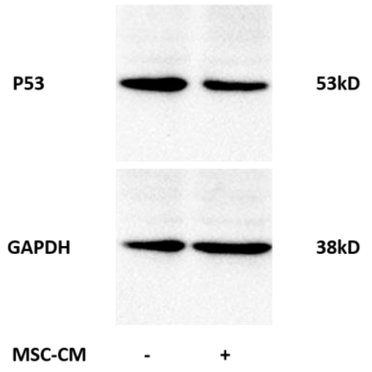
P53
53kD
GAPDH
38kD
MSC-CM - +

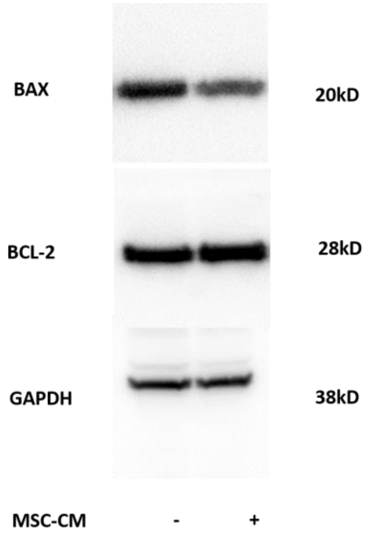
BAX
20kD
BCL-2
28kD
GAPDH
38kD
MSC-CM - +

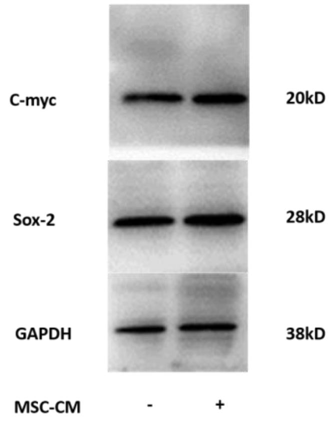
C-myc
20kD
Sox-2
28kD
GAPDH
38kD
MSC-CM - +

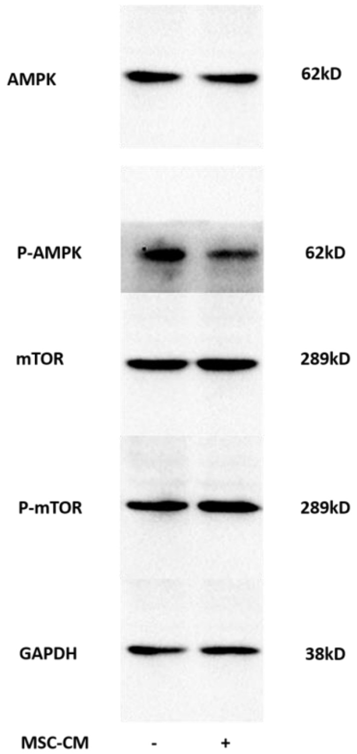
AMPK
62kD
P-AMPK
62kD
mTOR
289kD
P-mTOR
289kD
GAPDH
38kD
MSC-CM - +

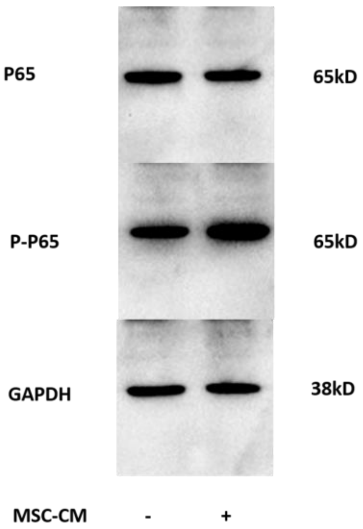
P65
65kD
P-P65
65kD
GAPDH
38kD
MSC-CM - +

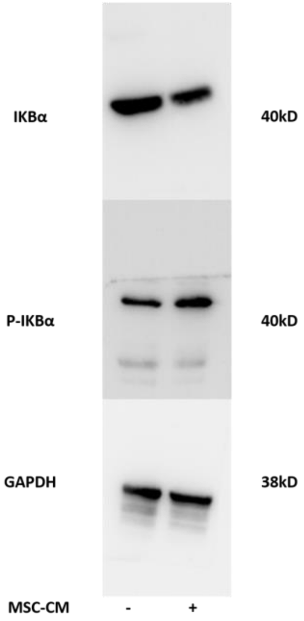
IKBα
40kD
P-IKBα
40kD
GAPDH
38kD
MSC-CM - +

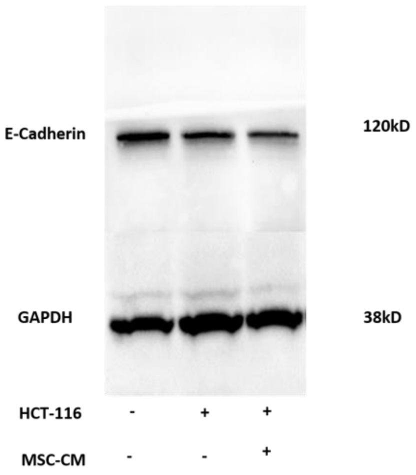
E-Cadherin
120kD
GAPDH
38kD
HCT-116 - + +
MSC-CM - - +

Supplementary Figure S2. Full-length blots of status of P53, Bax, Bcl-2, c-myc, Sox-2, E-Cadherin P65 and AMPK, mTOR, P65, IkBα phosphorylation in HCT-116 after treated with MSC-CM
